# Stomatoscope

**Published:** 1866-09

**Authors:** 


STOMATOSCOPE.—Among other novelties noticed in the *Med. Times and Gaz.*, is "a new instrument, to be termed the *stomatoscope*, exhibited last week to the Paris Surgical Society, by its inventor, Professor BURNS, of Breslau. A platinum spiral wire (inclosed in a box-wood cup, to prevent the transmission of heat,) brought to a red heat by the passage of an electric current from two of MIDDLEDORPS' elements, is placed in the mouth behind the teeth. The light reflected by a very small mirror is sufficiently intense to render the jaw transparent, so as to allow of the vessels proceeding to the roots of the teeth, the smallest specks of caries, etc, becoming visible. By reason of the transparency, the labial coronary artery may in some subjects be seen at the level of the commissure, and its course followed. The instrument is therefore likely to form a useful means of exploration in dental affections."

